# A perspective on Councils on Chiropractic Education accreditation standards and processes from the inside: a narrative description of expert opinion: Part 2: Analyses of particular responses to research findings

**DOI:** 10.1186/s12998-019-0276-5

**Published:** 2019-09-12

**Authors:** Stanley I. Innes, Vicki Cope, Charlotte Leboeuf-Yde, Bruce F. Walker

**Affiliations:** 1 College of Science, Health, Engineering and Education, Murdoch University, Murdoch, Australia; 2 Institute for Regional Health Research, University of Southern Denmark, DK-5000 Odens, Denmark

**Keywords:** Accreditation, Chiropractic, Competence, Qualitative, Council on chiropractic education, Vitalism

## Abstract

**Background:**

This is the second article reporting on a study that sought the views of people with extensive experience in Councils on Chiropractic Education (CCEs) on research that has raised concerns about variability in accreditation standards and processes for chiropractic programs (CPs) and chiropractic practice in general.

**Methods:**

This qualitative study employed in-depth semi-structured interviews that consisted of open-ended questions asking experts about their thoughts and views on a range of issues surrounding accreditation, graduate competency standards and processes. The interviews were audio-recorded, and transcribed verbatim in June and July of 2018. The transcripts were reviewed to develop codes and themes. The study followed the COREQ guidelines for qualitative studies.

**Results:**

The interviews revealed that these CCE experts were able to discern positive and negative elements of the accreditation standards and processes. They were, in general, satisfied with CCEs accreditation standards, graduating competencies, and site inspection processes. Most respondents believed that it was not possible to implement an identical set of international accreditation standards because of cultural and jurisdictional differences. This was thought more likely to be achieved if based on the notion of equivalence. Also, they expressed positive views toward an evidence-based CP curriculum and an outcomes-based assessment of student learning. However, they expressed concerns that an evidence-based approach may result in the overlooking of the clinician’s experience. Diverse views were found on the presence of *vitalism* in CPs. These ranged from thinking vitalism should only be taught in an historical context, it was only a minority who held this view and therefore an insignificant issue. Finally, that CCEs should not regulate these personal beliefs, as this was potentially censorship. The notable absence was that the participants omitted any mention of the implications for patient safety, values and outcomes.

**Conclusions:**

Expert opinions lead us to conclude that CCEs should embrace and pursue the widely accepted mainstream healthcare standards of an evidence-based approach and place the interests of the patient above that of the profession. Recommendations are made to this end with the intent of improving CCE standards and processes of accreditation.

**Electronic supplementary material:**

The online version of this article (10.1186/s12998-019-0276-5) contains supplementary material, which is available to authorized users.

## Background

This is the second article reporting on a study exploring accreditation standards and processes of chiropractic education. The role for the training of chiropractors is undertaken by Councils on Chiropractic Education (CCEs), which oversee the regulatory and educational standards for chiropractic education providers. These standards are outlined in their written documents. They are composed of a description of the competencies a student is expected to attain before graduation as well as a set of requirements for chiropractic programs, among others, curriculum content, facilities, and staff.

Previous studies have raised concerns in a number of areas of the accreditation processes conducted by CCEs [1–5]. These have included variability in standards between accrediting agencies [6], lack of an evidence-based (EB) approach [2], inclusion of non-evidence-based philosophies such as *vitalism* and *subluxation theory* [3]. Also, non-evidence-based beliefs have been identified in chiropractic students, who are resistant to the educative process [7], as well as deficits in their understanding of non-indications for care [5]. Finally, a relationship between chiropractic student personality traits and their clinical decisions has been demonstrated [4]. These issues probably reach beyond pedagogy and have implications for patient safety, quality of care and workforce mobility [8–15].

In Part One, the opinion was sought on each of these matters from CCE experts, as they may have valuable insights into CP regulatory matters and could corroborate and improve our understanding of the complexities of these concerns, as well as suggesting possible solutions (Part 1, Innes et al., in press). A qualitative approach was taken, because past research exploring these themes encountered an unexpected reluctance to respond to a survey [16], and a qualitative methodology facilitates the exploration of complex phenomena like this [17, 18].

When the CCE experts were asked for their views on these concerns, six common themes emerged across the five issues listed above. These were CCEs organizations had to negotiate a diverse profession with strongly held views that frequently resulted in conflict and they had to do this with limited resources. The respondents believed chiropractic should be integrated within the healthcare community, but efforts should be made to preserve its uniqueness. Concerns were expressed by respondents that profit motives often drove chiropractic program behaviours, and there was a wide range of views on how best to assess chiropractic programs for accreditation. These themes were over and above the interview questions and warranted separate reporting and discussion.

In this Part Two article we report on the responses of the CCE experts to each of the concerns posed in the interview questions and attempts to garner the diverse discussion and controversial professional responses found.

### Aim

The primary aim of this study was to explore the experience and beliefs of CCE experts of (re)-accreditation standards and processes of CP by seeking their views on the following issues:

I. All CCEs should perhaps implement an identical international set of competencies for all chiropractic students to achieve before graduation.
II. All CCEs should implement an identical set of accreditation and re-accreditation standards for CPs. This would include minimal staff qualifications and student hospital placements.
III. The processes and standards of site inspection teams of CPs.
IV. CCEs should watch over CPs to ensure students learn important course material. For example, learning the appropriate contra / non-indications for chiropractic care or helping students and CPs educators understand how student personality, attitudes, and beliefs may impact on clinical decision making.
V. Vitalism and evidence-based practice in CP course material.

## Method

This was a qualitative study utilizing in-depth semi-structured interviews in-person via Skype or telephone. The derivation of the questions has been detailed in the first study (Part 1, Innes et al., in press) and the interview questions (*aide de memoire*) are attached in Additional file 1. Ethics approval was obtained from the university Human Research Ethics Committee (2018/055) before recruitment and data collection.

### Participants and recruitment

Nine expert participants were recruited from thirteen email approaches. The full details of CCE member sample size, recruitment, consent, and confidentiality management are detailed in the first study (Part 1, Innes et al., in press). Two key representatives were sought from each of the 5 CCEs. The final sample consisted of nine participants (6 men and 3 women) who had an average of 14 years-experience with at least one CCE, two of whom were non-chiropractors. The interviews were conducted from May to July of 2018 and lasted between 32 and 62 min, with an average duration of 44 min.

### Data collection

Data were collected from consenting participants using a semi-structured in-depth interview process via Skype or telephone, because the respondents were located at a distance, both nationally and internationally.

The principle researcher conducted the interviews (*n* = 9). The nine participants were provided with the interview questions, generated from previous research findings, prior to the interview and invited to reflect on the questions. Participants were invited to make further comments as they felt appropriate to the topics under discussion. An *aide de memoire* was used to ensure consistency across all the interviews (Additional file 1).

### Data analysis

The data analysis is also detailed in Part One (Part 1, Innes et al., in press) but in summary the issues of trustworthiness of data and interpretation of the study required addressing credibility, transferability, dependability and confirmability [19]. The transcriptions were returned to the interviewees for verification of accuracy to increase credibility. The interviewer was familiar with relevant CCE documentation [1–3, 20]. This helped ensure credible interpretation of the interactions with the participants, thus improving methodological rigour [21]. To attain dependability and confirmability of the data, the analysis process (using NVivo 11 software) as outlined by Braun and Clarke [22],was reviewed by another qualitative expert. The interviews were reviewed by the lead researcher and discussed with a qualitative research investigator, until they agreed that thematic saturation had been reached. They agreed this occurred after the ninth interview. Implications of these findings were discussed and a list of recommendations compiled.

## Results

The CCE experts, when responding to the possibility of implementing identical international CCE standards, stated that their views were the same for both the expectations for students’ graduation competencies and the written accreditation standards for CPs. Consequently, the graduate competencies and accreditation standards findings were grouped together.

### Standards for competencies of graduating chiropractors and accreditation

#### Suggestions for changes to improve the domains and subdomains of CCEs standards?

Six of the nine interviewees could not think of any changes to the domains and subdomains of their respective CCEs standards for the improvement of graduate competencies and accreditation standards. One third of the nine felt that the ‘real issue’ was how to facilitate CPs to want to seek compliance rather than be forced to achieve a set of standards. All respondents spoke of the inherent ambiguity in language. That is, one word may have different meanings for different cultures or societies. Consequently, the respondents spoke of the need for more work on definitions and on the terms commonly used in accreditation standards to resolve this lack of clarity. The words most often cited were “chiropractor” and “diagnosis”. The possession of more detailed definitions was thought to result in an increased ability to assess CPs as well as to create a more portable international workforce.

R2: “I don’t think actually it’s an issue of improving the standards per se. As it is to get compliance. And I think that’s the bigger issue”.

#### Is it possible to create identical international standards?

The task of achieving identical international standards was seen to be unachievable because of cultural differences and local jurisdictional variations. It was thought that a more appropriate expression was “equivalence of standards”. To this end three participants thought that it would be helpful if a core set of standards was created.

R1: “Absolutely not. The word is not identical competencies but equivalent competencies. We - again at the CCE – we struggled with that a whole lot and I think there needs to be core standards that are much the same across the board and across the world”.

This sentiment appears to be at odds with the thoughts of three other respondents who believed that even trying to achieve something fundamental, such as a definition of “chiropractor”, was highly unlikely.

R2: “Good luck with that (*sic defining chiropractic*) . . . . It’s a political issue rather than a clinical issue. And you know when you look at most of the studies on what chiropractors do, most of what they do is neuromuscular skeletal problems”.

#### Views on an EB approach to the formation of accreditation standards and processes

All CCE respondents acknowledged the importance of an EB approach to the formation of accreditation standards. One expressed the view that the entire healthcare community is adopting an EB approach to education and practice wherever possible. Therefore, it is nothing more than what should be expected of CCE’s. However, five of the nine respondents added caveats, such as, there is no evidence for everything a chiropractor does and the practitioner’s clinical experience is an important consideration in accreditation standard development. Two thirds of the CCE representatives thought that research into these standards was needed and that CCEs were strongly positioned to guide and inform it. However, it was contended that CCEs were under-resourced to do this research themselves.

R1: “To have a strictly evidence-based practice is probably not necessary and probably a hindrance in that you’ve got to keep yourself from using stuff that is truly valuable”.

### The process and standards of site inspection teams of CPs

#### Views on the ability of site inspection teams to monitor CP compliance

There was widespread but conditional agreement that site inspection teams formed a valuable part of the monitoring process of CPs. At least one third of the respondents thought that important issues were obtaining team members who had the necessary personal qualities, such as interpersonal and critical thinking skills. Additionally, team members were deemed to require an understanding of the accreditation standards, be experts in their field, and have prior experience. Further, the teams themselves should be well resourced, carefully trained and led by a skilled leader. Two respondents commented that there was only a small pool of chiropractors available to choose from for this task and that more consideration should be given to including experts from outside the profession.

R5: “My view is that the training of the team members, generally speaking, is not very good. They need to be prepared to process lots of information. Some of the team members are not very good at this. They need to know how to collect data and interpret it. In other ways most of the team members tend to struggle with this task. They need to have critical thinking skills to be able to do all this. It is very difficult and involves a lot of training”.

Respondents also thought that inspection teams needed to be able to see through CPs that submitted “glowing” self-evaluation reports or attempted to hide deficiencies. Respondents also spoke of the importance of pre-inspection knowledge or intelligence from sources outside of the CP self-evaluation report (students, staff, and professional chiropractic association members).

#### Views on the ability of site inspection teams for quality improvement of CPs

Approximately half of the participants thought that carefully constructed experienced teams, which have developed a strong rapport with CPs were an important source of thoughtful and meaningful suggestions for continued improvement. Many respondents thought that site teams offered CPs located within a university setting a means for leverage to bring about changes with the threat of the removal of accreditation. Possible changes mentioned were more full-time staff, funding and removal of unnecessary curriculum requirements.

R6: “So I think it could go both ways, but I’ve seen many examples where the site evaluation report has been instrumental in making improvements. And also provided the impetus especially if they’re part of universities. So, they have this report, now this professional team’s accrediting body has said we need to do this. And that gives them ammunition. It’s not just the faculty or the management of the programme saying”.

#### Should final site inspection teams’ reports be made public?

Respondents were either strongly in favour of transparency or thought it was a “conundrum”. Reasons for publishing findings of CP site inspections included; exposure of bad behaviour forced change, the public has a right to know, it is standard practice around the world and in other health professions, and CPs are resourceful and can manage the stress of adverse findings. Reasons against disclosure were; it is distressing for CPs and can damage their image, confidentiality is a facilitator of open and frank disclosure by CPs to CCEs and the public cannot understand the complexities around (re) accreditation processes. One CCE expert reported no adverse effects from publishing the site inspection team’s final report on their website and could see no reason for others not to do so. Finally, several respondents thought that there is not uniformity in site team evaluations and this would mean that disclosure of inequitable levels of scrutiny was unfair for CPs.

R9: “But if they think that it’s going to be divulged to the public there’s so much competition that is out there for students - you know there are unscrupulous institutions, universities, CPs, you name it, that would use public information to damage the reputation of another programme ... And so if these - if the self studies were to be made public I think you would end up with them being much more benign, whitewashed, lacking some of the critical appraisal that we encourage institutions to have when they’re writing their self studies.”

### Factors relevant to ensuring students learn important course material

#### Should CCEs ensure students learn core material e.g. contra/non/indications for care?

One third of respondents thought that it was necessary to make sure students know core material and that this warranted inclusion as a formal accreditation standard. However, half thought that this was likely to be contentious because of the lack of agreement on what core material would likely be. For example, there is a diversity of opinions on the reasons for spinal manipulation. Some groups of practitioners would believe that regular spinal manipulation prevents a range of non-musculoskeletal conditions, while others would see it as providing short-term pain relief. Consequently, the indications for spinal manipulation are better not prescribed.

R1: “But in as much as we feel that a chiropractic adjustment given from time to time or regularly has preventative value how do you measure something that you’ve prevented. . . . . . . This is the argument that would float around the table if we were in an accreditation setting.”

#### Should there be minimal faculty qualifications?

Two thirds thought that the difficulty with seeking faculty with high levels of academic qualifications, such as a PhD, was that it might preclude good teachers who have clinical experience. Many held reservations that a highly academically qualified person was not necessarily a “good” teacher with sound pedagogy. One expressed the view that mandating highly qualified staff imposed a much higher wages cost for CPs.

R3: “*In other words, an instructor may hold a PhD but have no teaching credentials or competencies, which would not optimize the educational process*”.

#### Should chiropractic students have a hospital placement experience?

Almost half of the participants believed hospital experience would be an important step for integration of chiropractic into mainstream health care. This experience was seen as improving communication between mainstream healthcare and chiropractic as well as enhancing student diagnostic skills. Two CCE responders thought that the benefits did not outweigh the likely cost and difficulty of arranging placements.

R7*: “*It’s the only way forward to get inter-disciplinary understanding of the profession and transfer of knowledge between professions*”.*

#### Should students be taught insight into their own personality?

One third of respondents commented that they had not thought of personality as being a factor in clinical decision making. The remainder thought that, although likely a factor, would not warrant inclusion as a consideration in a formal statement in accreditation standards.

#### What is the CCEs role in chiropractic students’ non-evidence-based beliefs?

Of all respondents, six stated that non-evidence-based beliefs of students should be dealt with by CPs teaching an EB approach and / or greater critical thinking skills, or as R3 stated “*how to learn, not what to learn*”. Other suggestions included open debate about the curriculum regarding these issues, employing more faculty that are EB, and that this is a post-graduate continuing education issue rather than a requirement of an accrediting agency. Two respondents felt that it did not warrant a dedicated accreditation standard. Finally, one CCE informant felt that the phenomenon of non-evidence-based beliefs warranted careful exploration as to their origin and proffered that solutions most probably lay in regarding them as being analogous to religious beliefs.

R6: “But it needs - yeah we need experienced faculty. Evidence based faculty and then approach it that way.”

### Evidence-based approaches and vitalism

#### Your thoughts on vitalism in CPs?

Half of the respondents thought that *vitalism* in chiropractic education was an impediment for the integration of chiropractic into mainstream health care. Two respondents stated that it was too difficult to write standards to prescribe against *vitalism* being taught other than in a historical context. Interestingly, one responded refused to comment on this question.

R7: “Because we’re seeing institutions graduating with a vitalistic model which is inconsistent with modern healthcare. And the reason they’re being accredited is because they’re ticking all the boxes. . . . On the one hand you’re teaching them (students) everything that’s evidence based. It’s physiology, it’s biology, it’s psychology. All the things that we have good evidence for understanding. And then we’re saying “And then you’ve got magic.” And those two things don’t mix very well. . It’s spoken and not written. . . . . And policing that is difficult.”

Some suggested it was better to do nothing and, to use the words of R1, “*turn a blind eye*” to *vitalism,* as it is always going to be there, is only championed by a minority of practitioners, and was too hard to deal with.

In contrast, two other respondents thought that the role of the CCE was to act as an accreditor and not a regulator. By acting as a regulator CCEs activities would likely result in inappropriate censorship.

R3: “The devotion to vitalism or other theories falls under the doctrine of “academic freedom”. Students and instructors should remain unrestrained in their pursuit of ideas and theories. Accreditation has no role in deciding which theories or beliefs are included in course material.”

#### Your thoughts on an EBP approach in accreditation standards for CPs?

One third of participants thought that there was insufficient emphasis placed on an EB approach to education and practice.

R1: *“It (EBP) should be everywhere. Especially in patient care. It should be in lights.”*

Four of the nine responders expressed reservations about a “totally” EB approach. Concerns were voiced that this involves a heavy emphasis on randomised-controlled studies, which may not always be applicable in specific instances. This in turn was thought to lead to the stopping of many helpful chiropractic techniques because of the lack of any supportive evidence. Finally, an EBP approach was thought to likely result in a reduction of the importance of the practitioners’ clinical experience and knowledge. No respondents mentioned patient preference, values or safety as a consideration.

## Discussion

### Summary of findings

The interviews revealed that respondents were, in general, satisfied with CCE accreditation standards, graduating competencies, and processes. They did not believe it was possible to implement an identical set of standards across all CCEs because of cultural differences. Rather, they stated that it would be better to create a core set of standards that were approximately the same or equivalent and these would require clear definitions of key words such as “chiropractic” and “diagnosis”.

Mixed views were expressed on the making public of final site inspection team reports of CP accreditations. Teaching skills and clinical experience of academic staff were valued at least as highly as attaining higher qualifications such as a PhD degree. A hospital placement for students was seen as offering a means to better integrate chiropractic into mainstream health care.

While respondents thought favourably of using an EB approach to accrediting CPs, they expressed some reservations that this might lead to the loss of valuable aspects of chiropractic practice, for which there is an absence of evidence. CCE experts thought that the most appropriate way to deal with students’ non-EB beliefs was for the CP to be evidence-based. Finally, there were mixed views on the presence of *vitalism* in CPs. Half of the respondents thought that the teaching of vitalism in CPs was an impediment to the integration of chiropractic in mainstream healthcare and that it was very difficult to police in CPs. It was also stated that so few held this view that it was probably not worth the effort of writing prohibitive standards. Others thought that *vitalism* was a matter of academic freedom, and accreditation has no role in deciding whether this should be included in CP curriculum.

### General discussion of implications

#### Standards for competencies of graduating chiropractors and accreditation

##### Suggested improvements to CCEs standards & identical international standards?

The respondents were generally satisfied with the existing accreditation standards and processes. The CCE experts thought that improvements would come from increased engagement by CPs in accreditation processes that should be constructed around a clearly defined and assessable set of core equivalent standards rather than identical standards.

This view of the CCE experts resonates with current research investigating medical accreditation that has identified as a major challenge to reforms is the lack of a common understanding of the terms and words used by stakeholders [23]. The lack of clarity of language negatively impacts on the engagement of all the stakeholders, creation of a shared agenda, establishing of goals, and methodologies for evaluating changes [23]. As such, definitions are of great importance and, therefore, become the starting point for reform [24, 25].

Part One of this study found that current CCE accreditation standards do not contain detailed definitions and standards, because they have to accommodate a diverse range of intra-professional views, such as *vitalism,* in CPs [5]. Part Two adds further detail, by finding that it is also due to the downplaying, or even refusal to comment, because of the difficulty in writing standards for a minority issue. This suggests that the creation of a clearly defined set of standards will be an unlikely event as the respondents in this study saw great difficulty in arriving at an agreement on the fundamental issues, such as a definition of “chiropractic”.

##### Views on an EB approach to the formation of accreditation standards and processes

There is a recognition by the participants in this study and chiropractic educators of the need to improve the quality of chiropractic all over the world with global consistency in accreditation and assessment of chiropractic education [26]. Medical education has demonstrated that this is possible, when it is founded on a scientific and EB approach to the clinical sciences and practice [27]. Previous studies found that the uptake by CCEs and chiropractic practice in general of an EB approach has been slow and incomplete [2, 28, 29], and this also appears in the response of some participants in this study, who wanted to defer to clinician experience as the most important factor in making clinical decisions. It is the authors’ contention that chiropractic internationally should wholeheartedly embrace this approach. The basing of the entire chiropractic curriculum upon an EB approach would require a substantive effort and consensus among CCEs to allow for updating of their accreditation standards and afford the opportunity of becoming more relevant to twenty-first century chiropractic practice.

Some suggested areas of scrutiny would be the number of hours required for courses in X-ray physics and positioning in an era where clinical practice guidelines around the world are advising against the routine use of spinal x-rays, the replacing of chiropractic philosophy courses with increased hours in understanding and interpreting research evidence, and teaching students how to run solo / private practices, where the focus will be on motivating and sustaining behavioural changes to manage chronic health co-morbidities associated with persistent or recurrent spinal / musculoskeletal conditions such as obesity.

#### The process and standards of site inspection teams of CPs

The respondents in this study expressed confidence in the site inspection process to monitor and apply accreditation standards. However, research has found that site inspections are of unknown reliability [15], under-investigated [30–32], the teams are often poorly trained [33], poorly selected [34] and in need of a standardised report structure for a comprehensive assessment measured against the accreditation standards [31]. This raises the concern that the confidence expressed by the CCE experts in the site inspection processes may have been ill-founded. For example, a lack of standardization of site inspection is seen by the presence within the same CCE of CPs who openly adopt a *vitalist* focused curriculum [35] and those who have signed a declaration that *vitalism* has no place in the modern curriculum, as the belief that it is the cause of disease is unsupported by any type of evidence [36]. A move toward transparency by making final site inspection team reports public may create greater accountability and explain how this heterogeneous situation can exist.

CCE experts in this study viewed site inspection teams as an important lever for quality improvement. This monitoring and reviewing role places them in a position to facilitate the introduction of identified innovations to teaching found in recent research, such as the impact of students’ personality on their clinical decisions [4]. Consideration may also be given to the common practice in academia of the inclusion of colleagues who come from different professional or academic backgrounds that often brings different and insightful points. Another common practice worthy of consideration, as the consumers of the education under review, is the obligatory inclusion of student reviewers as members of inspection teams [37]. In addition, they could also address deficiencies like inadequate case mix in chiropractic teaching clinics [38] by helping CPs explore hospital placements, also recently shown to address this issue for chiropractic students [39]. To this end CCEs could develop a core standard for clinical competency that encourages CPs to provide greater interprofessional clinical training opportunities.

#### Factors relevant to ensuring students learn important course material

##### Should CCEs ensure students learn core material e.g. contra/non/indications for care?

The preference to be accommodating of diverse views was also seen in the reluctance of CCEs experts to mandate that chiropractic students know the contra/non/indications for spinal manipulation. Contemporary research is calling for the cessation of non-indicated care or low value care as an important measure to reduce the financial burden of low back pain on societies [40, 41]. On the surface, the primary concern appears to be consideration of the profession. An EB approach would see the three aspects of research evidence, clinician expertise and patient values and circumstances as the drivers for such decisions [42]. We contend that the application of this framework would yield a different response to the importance of CCE standards appropriately and adequately ensuring the learning of core material.

##### What is the CCEs role in chiropractic students’ non evidence-based beliefs?

The experts in this study were unsurprised at the presence of non-evidence-based beliefs in chiropractic students. The almost unanimous view was that this issue was best dealt with by the CPs taking an EB approach. If this was an effective strategy, then logically it should follow that levels of chiropractic student non-EB beliefs would decrease over the duration of the CP. This was not the case in two Australian university-based chiropractic programs [7]. The most significant influences in these students’ beliefs and attitudes were external peers and their past experiences [43].

This phenomenon has also been studied in osteopathic treatment in Britain [44]. Here, osteopathic ‘philosophy’ was found to be seen as superior to science and distorted the way practitioners and students view, judge and reject the results from research evidence and guidelines. This ‘lens’ elevates the value of personal experiences, anecdotes and the teachings of ‘expert’ therapists, above results from systematic reviews and meta-analyses.

This offers a possible explanation of why the presentation of evidence does not change non-EB beliefs in chiropractic students. It is overly simplistic to think that EB content alone will ensure that students will graduate without non-EB beliefs. Rather we are swayed by the respondent in this study who thought that investigations for solutions be focused on regarding non-EB beliefs as being analogous to ideological or indeed religious beliefs.

##### Should there be minimal faculty qualifications?

Respondents commonly expressed the view that clinical and teaching expertise and research training (such as a PhD) in chiropractic educators are in some way mutually exclusive. We suspect the finding that medical faculty with outdated knowledge of research methodologies, poor skills in critical evaluation of medical information were barriers to the adoption of EB medicine would also apply to chiropractic faculty [45, 46]. If chiropractic education and the profession is to continue to establish itself as a credible and mature health profession, then there needs to be an emphasis on not just training semi-skilled consumers of research but also on facilitating increased numbers of post graduate researchers and research active academics within chiropractic programs [47, 48]. CCEs standards may include expectations toward this end. Also this could include courses in adult learning and pedagogy for chiropractic faculty to address reservations that having a PhD does not make one a “good” teacher. Also, CCEs standards could encourage the CPs to hire faculty with advanced degrees in education.

#### Evidence-based approaches and vitalism

##### Your thoughts on vitalism in CPs?

CCEs are in a powerful position to embed an EB approach into educational providers’ curricula and to improve patient outcomes as well as further align chiropractic with accepted healthcare standards [49–52]. Many chiropractic educators do not believe that students should be learning how to conduct their own original research. Instead, they need to learn how to become good “consumers” of research evidence [2]. It may be possible for CCEs to agree on a minimum number of courses about research evidence in the chiropractic curriculum that teach chiropractic students the principles of acquiring, appraising and applying research evidence in clinical practice. This could be further scaffolded with the teaching of an understanding of how systematic reviews are conducted and clinical practice guidelines are developed.

The authors agree with the respondents in this study, who view the role of the CCE as providing peer view of the CP educational processes with an emphasis on what is best for the student. However, the authors respectfully disagree that this is their sole remit and that *vitalism* falls outside their working brief. Non-EB beliefs or philosophies have implications for patient safety and quality of care and should be a CCE consideration. In Canada non-EB beliefs have shown to be associated with high levels of anti-vaccination attitudes, use of non-evidence-based treatment choices, non-guideline use of X-rays and low levels of inter-professional collaboration [6]. Further, in a group of people, conscious of protecting the chiropractic profession, a critical approach to non-EB and overenthusiastic clinical approaches might have a stronger protective effect when dealing with such issues. Also, we are cognizant that there are other professional regulatory bodies that act to protect the health and safety of the public and that this task does not rest solely with CCEs.

The recent WFC education conference delegates statement [26] echoed the need for CCEs to become also regulators, with the public’s safety at heart. In addition to the questions of research evidence and clinician expertise, CCE organizations need also ask themselves the bedrock question of their accreditation standards and processes “Does it make care better for patients?” [53].

### Strengths and limitations

The strengths and limitations of this study have been discussed in the article titled A perspective on Councils on Chiropractic Education accreditation standards and processes from the inside: A narrative description of expert opinion. Part 1: Themes. In brief, this was a qualitative study with few participants, meaning that the findings are not representative of the views of all members of all CCEs internationally. Consideration should also be given to the possibility of community bias. However, the sampling had been designed to garner views from experts within all CCEs and 9 of 12 CCE experts accepted participation. The CCE participants had an average of 14 years’ experience, which caused us to be confident they have provided a rich insight into the issues surrounding CCE matters. Anonymity probably ensured honest and open answers and the responses were in line with concepts already encountered in previous surveys and in personal communication with this type of persons. Also, the authors are confident they have addressed the issues surrounding rigour in qualitative research through reflexivity [54], credibility, transferability, dependability and confirmability [21].

### Recommendations

As in Part One of this study, the interviews with CCE Experts has raised several issues and, based on these as well the available literature, the authors make a number of recommendations (Table 1), in particular, the concerns about variability in accreditation standards and processes for chiropractic programs (CPs) and chiropractic practice in general.

**Table 1:** Summary table of recommendations

	Recommendation	Justification
1.	Creation of an internationally acceptable set of equivalent accreditation standards and processes	For greater public confidence, graduate chiropractic homogeneity and workforce portability.
2.	An EB approach be adopted for accreditation standards and processes.	Facilitate the integration into mainstream health care.
3.	Standardized inspection team member selection, training and format for reporting.	Improve the quality of CP assessment and quality improvement processes for improved educative processes.
4.	Broaden the scope for site inspection team composition e.g., students, academic colleagues	Gain broader insights into the issues facing CPs and their possible solutions.
5.	Facilitate research to explore the optimal mix between an outcomes-based and prescriptive (hybrid) approach to the competency levels of graduating chiropractic students.	This will develop, inform and improve accreditation standards.
6.	Make site inspection team reports public.	This is the broader societal expectation and will align chiropractic with the mainstream standards of transparency.
7.	Move toward minimum faculty qualifications of a PhD.	This would improve the educational standing of CPs and enhance research capability and quality.
8.	CCEs standards may include expectations for courses in adult learning & pedagogy for chiropractic faculty.	This would address reservations that having a PhD does not make one a “good” teacher.
9.	CCEs standards could encourage the CPs to hire faculty with advanced degrees in education.	To scaffold the teaching quality of CPs to improve student learning outcomes
10.	Provide student hospital placements	Improve graduate student quality and interdisciplinarity skills.
11.	Develop a core standard for clinical competency that ensures a meaningful student clinical training experience	Graduates are better prepared to engage in safe and effective practice.
12.	Investigate innovative dimensions of student clinical decision making such as personality type.	Improve graduating students’ clinical decision-making skills.
13.	Address unorthodox (vitalism and ‘subluxation’) practice patterns in CCE accreditation standards.	Align chiropractic education with contemporary EB approaches to health profession education.
14.	The development of a core standard for literacy in critical thinking	This would result in an increased ability to consume research evidence and translate this into practice for improved patient outcomes.

The intent is for the tables from Part One and Part Two to complement each other and not repeat common issues. These recommendations are intended to create a uniform high standard of practitioners who are more likely to be in accord with the mainstream healthcare standard of an EB approach across all CCE-controlled regions. This would ensure and safeguard the international trust in chiropractors’ ability to deliver ethical, safe and valid care across and within international borders.

## Conclusions

The overarching aim of this and the previous study (Part 1, Innes et al., in press) was to explore the experience and beliefs of CCE experts about variability in accreditation standards and processes for chiropractic programs (CPs) and chiropractic practice in general as well as making recommendations for improvements.

We found that when experts are queried about the ‘inner life’ of the CCEs, they can discern between positive and negative elements in the CCEs procedures. They can also explain, in an understandable way, the difficulties encountered in determining the aims, objectives of the CCEs standards and also in the actual execution of the accreditation process.

However, there was a considerable diversity of opinions on many topics.

We interpreted the reasons for the considerable variability between chiropractic programs worldwide to be embedded in a political negotiation process of CCEs determining their standards. The result has been a polite acceptance of ‘philosophical’ or “ideological” views of some chiropractors. In other words, the group of chiropractors, who favour mainly a musculoskeletal approach, co-habitats with those chiropractors who believe that chiropractic treatment also has an effect on a range of non-musculoskeletal conditions. This results in standards and procedures that are sufficiently non-specific to allow for both types of institutions to pass the CCE accreditation requirements.

This has real world implications. From a public health perspective, chiropractors who are practicing from a ‘philosophical’ perspective (non-EB) are more likely to prevent the adoption of chiropractors in the mainstream medical world.

We argue that the “raison d’être” of CCEs is not to solely oversee a chiropractic education that encompasses all understandings of chiropractic practice. We recommend that the key question for accreditation bodies is “Does it make for better patient care?” and we call on CCEs to take a stand and better serve the patients’ best interests and not the conservative chiropractic profession.

To this end we have made recommendations that include CCEs embracing and pursuing an EB approach, which in the end will place the interests of the patient above that of vested segments of the profession.

## Additional file

Additional file 1: Interview Questions. (DOCX 17 kb)

## Data Availability

Not applicable.

## Abbreviations

CCE: Council on Chiropractic Education
CP: Chiropractic Program
EB: Evidence-based
