# Supplementary material for: A perspective on Councils on Chiropractic Education accreditation standards and processes from the inside: a narrative description of expert opinion: Part 2: Analyses of particular responses to research findings
Source: Chiropr Man Therap. 2019 Sep 12;27:56. doi: 10.1186/s12998-019-0276-5 (PMC6739975; doi:10.1186/s12998-019-0276-5)
Supplement: Supplementary file 1 — Interview Questions. (DOCX 17 kb) [file 12998_2019_276_MOESM1_ESM.docx]

# Additional File 1

Title: Aide de Memoire / Interview questions.

*Opening questions*:

“Can you tell me generally about your involvement with the CCE?”

“Can you tell me what you see as the challenges for CCEs improving the standards of Chiropractic education?”

## Sub-question 1.

*Open-ended question:* What are your views about implementing identical graduating chiropractor competency standards for all CCEs?

Is there anything you would like to change in the domains of competencies for graduating chiropractors?

*Prompts:* Could you talk more about ...? Can you explain that more?

Prompts that relate to previous studies would include – “In our research to date we have found or concluded that

- There is a need for an appropriate definition of a chiropractor
- There is potential for ambiguity in the use of terms between CCEs
- There is a need for common domains for all CCEs
- There is a need evidence based knowledge content in competencies
- There is insufficient emphasis placed on ethical and professional practice behaviours
- involvement in research exploring optimal structures and educative processes that produce the required competencies.

Do you have any comment on this finding or conclusion?

*Concluding question*: That’s all I would like to ask, is there anything else you’d like to talk about or ask me regarding this issue?

## Sub-question 2.

*Open-ended question:* What are your views about implementing identical accreditation standards for all CCE?

Is there anything you would like to change in the domains of accrediting standards for CPs?

*Prompts:* Could you talk more about...? Can you explain that more?

Prompts that relate to previous studies would include – “In our research to date we have found or concluded that:

- there is a need for a definition of education standards
- should there be a domain for distance education?
- all accreditation standards should be based on literature reviews
- the development of accreditation standards should take on board input from all stakeholders
- chiropractic students should (at least in part) have hospitals experience
- there should be minimal levels of qualifications for chiropractic faculty (e.g. industry standard of PhD),
- Chiropractic program (CP) curricula should be taught in a multimodal format
- There should be core material for all CPs
- CP mission statement should have a social responsibility
- There should be a minimal set of financial standards for all CPs

Do you have any comment on this finding or conclusion?

- *Concluding question*: That’s all I would like to ask, is there anything else you’d like to talk about or ask me regarding this issue?

## Sub-question 3.

*Open-ended question*: What are your views on the CCEs role in CPs to ensure that students learn relevant clinical course material? For example, learning the contra-indications for chiropractic care?

*Prompts:* Could you talk more about...? Can you explain that more?

Prompts that relate to previous studies would include – “In our research to date we have found or concluded that:

- non-indications for chiropractic care are poorly understood

Do you have any comment on this finding or conclusion?

*Concluding question*: That’s all I would like to ask, is there anything else you’d like to talk about or ask me regarding this issue?

## Sub-question 4.

*Open-ended question*: What are your views on CCEs requiring CPs to teach students about understanding their own personality, attitudes or beliefs and how these may impact on their clinical decisions?

*Prompts:* Could you talk more about...? Can you explain that more?

Prompts that relate to previous studies would include – “In our research to date we have found or concluded that:

- Intolerance of uncertainty impacts negatively on clinical decision making
- There are non-evidence based beliefs such as “SMT helps immune system, prevents disease in general, and prevents spinal degeneration” that suggests less than optimal care will be delivered by these students

Do you have any comment on this finding or conclusion?

*Concluding question*: That’s all I would like to ask, is there anything else you’d like to talk about or ask me regarding this issue?

## Sub-question 5.

*Open-ended question*: What are your views about the inclusion of vitalism into CP course material? What are your views on the inclusion of EBP into CP course material?

*Prompts:* Could you talk more about...? Can you explain that more?

Prompts that relate to previous studies would include – “In our research to date we have found or concluded that:

- There is no specific mention of Vitalism in CCE standards
- There is no specific mention of EBP in CCE standards.

Do you have any comment on this finding or conclusion?

*Concluding question*: That’s all I would like to ask, is there anything else you’d like to talk about or ask me regarding this issue?

Closing instructions:

“We would like to thank you for your time today. Are there any final comments you would like to make or any question you would like to ask? If you would like to learn about the results of this study then I am happy to send you an abstract of the final published study”.
